# Frequency of Non-ST Segment Elevation Myocardial Infarction (NSTEMI) in Acute Coronary Syndrome With Normal Electrocardiogram (ECG): Insights From a Cardiology Hospital in Pakistan

**DOI:** 10.7759/cureus.8758

**Published:** 2020-06-22

**Authors:** Rozi Khan, Junaid Akhter, Ussama Munir, Talal Almas, Waqas Ullah

**Affiliations:** 1 Internal Medicine, MedStar Union Memorial Hospital, Baltimore, USA; 2 Internal Medicine, Bolan University of Medical and Health Sciences, Quetta, PAK; 3 Cardiology, Tabba Heart Institute, Karachi, PAK; 4 Cardiology, Bahawal Victoria Hospital, Bahawalpur, PAK; 5 Internal Medicine, Royal College of Surgeons in Ireland, Dublin, IRL; 6 Internal Medicine, Abington Hospital-Jefferson Health, Abington, USA

**Keywords:** : acute coronary syndrome, non st-segment elevation myocardial infarction, electrocardiogram (ecg/ekg), cardiac troponin

## Abstract

Introduction

Patients presenting to the emergency department with a non-ST segment elevation myocardial infarction (NSTEMI) frequently have unremarkable electrocardiography (ECG) reports, alluding to the unreliable nature of ECG in diagnosing NSTEMI. This study aims to assess the burden of NSTEMI in acute coronary syndrome (ACS) with unremarkable ECG, elucidating that in patients presenting with acute retrosternal chest pain, NSTEMI should not be excluded unless cardiac enzyme levels are assessed.

Methods

All patients who fulfilled the inclusion criteria in the Department of Cardiology, Tabba Heart Institute, Karachi were included. After obtaining informed written consent, a detailed history was taken. Clinical examination was consequently performed, and an ECG, along with the cardiac enzymes implicated in ACS, such as troponin I, was evaluated. The proportion of normal ECGs in the context of an NSTEMI was duly noted.

Result

A total of 215 patients with ACS presenting within 24 hours of the onset of symptoms, on a background of unremarkable ECG reports, were included. One hundred thirty-eight (64.2%) were males and 77 (35.8%) were females, with the mean age being 54.3 + 7.6 years. A confirmed diagnosis of NSTEMI was made in 49 (22.8%) of the total cases.

Conclusion

The frequency of patients presenting with an NSTEMI within 24 hours of the onset of symptoms, and having normal ECG findings, was strikingly high in patients presenting to the Tabba Heart Institute, Karachi, Pakistan. These findings were more common in males and in older patients.

## Introduction

Coronary artery disease (CAD) is a global health problem of enormous proportions. It is the leading cause of morbidity and mortality in both men and women worldwide. In 2016, CAD was the leading cause of deaths attributable to cardiovascular disease in the United States (43.2%), followed by stroke (16.9%) and high blood pressure (9.8%) [1]. According to the American Heart Association, cardiovascular disease accounted for more than 17.6 million in 2016 and 17.5 million in 2014 deaths per year, numbers that are expected to rise to more than 23.6 million by 2030. According to the World Health Organisation (WHO), an estimated 17.5 million people die from cardiovascular diseases annually, representing 31% of all global deaths. Of these deaths, an estimated 7.4 million are due to CAD [1].

Acute coronary syndrome (ACS) is a syndrome consisting of signs and symptoms due to decreased blood flow in coronary arteries such that part of the heart muscle is unable to function properly due to hypoperfusion [2]. ACS is a medical emergency that warrants emergent intervention. ACS is mainly classified according to electrocardiography (ECG) findings on admission and the levels of cardiac enzymes, such as troponin and CK-MB. ACS may be further classified as either an ST-elevation ACS (STE-ACS) or a non-ST-elevation ACS (NSTE-ACS) in accordance with ECG findings and troponin T and I levels [3].

Patients with ST-elevation ACS present with acute chest pain and ST-segment elevation on ECG. Non-ST-elevation ACS patients, on the contrary, present with acute chest pain but without ST-segment elevation. ECG usually shows transient or persistent ST-segment depression or T wave inversion or flat T wave or no ECG changes at all. NSTE-ACS is further subdivided into either unstable angina (UA), with normal troponin levels, or non-ST segment elevation myocardial infarction (NSTEMI) with raised troponin levels [4].

Many patients with NSTE-ACS can have normal ECG findings on presentation in the emergency room, showing that ECG may be non-diagnostic in a multitude of ACS cases [5]. Patients presenting to the emergency department with chest pain who demonstrate a normal ECG have a low rate of morbidity and mortality from cardiac complications, but the possibility of an NSTEMI should not be ignored [6-8]. Every patient presenting with ACS and having normal ECG at presentation should be thoroughly evaluated, and troponin T and troponin I levels should be evaluated to rule out myocardial damage secondary to myocardial ischemia so that further cardiac complications can be detected and duly managed. The purpose of this study therefore is to ascertain the frequency of NSTEMI in ACS with normal ECG, gauging data and evidence that ACS with an unremarkable ECG can still have underlying myocardial damage as evidenced by raised Troponin I levels. The data on this topic remains scarce, with one study showing that the frequency of ACS in patients with normal ECG was 17% [7]. This study will further assess the burden of NSTEMI in ACS with normal ECG so that patients with chest pain and normal ECG should not be ignored without the evaluation of cardiac biomarkers.

## Materials and methods

Using a 5% margin of error and 95% level of confidence, the sample size was calculated by using the World Health Organisation (WHO) calculator [8]. Consecutive patients with NSTE-ACS presenting to the emergency of Cardiology Department, Tabba Heart Institute, Karachi, Pakistan, were considered. After obtaining permission from the ethical review committee, patients fulfilling the exclusion and inclusion criteria (detailed below) were selected. Informed written consent was obtained and history of the time of onset of symptoms, history of co-morbid conditions like diabetes, hypertension, smoking status, dyslipidemia with documentary evidence of the consumption of anti-lipidemic medication from consultant physician, were obtained. Clinical examination was performed thereafter along with the measurement of BMI and investigations (ECG and Troponin-I). The information was then filled in the proforma attached as Annexure. Serum Troponin-I levels after six hours of the onset of typical ischemic chest pain in the context of NSTE-ACS were measured. Patients in whom these levels were found to be greater than 0.5 ng/ml were declared as having NSTEMI and were managed as per the institutional guidelines.

Inclusion criteria

All ACS patients (as determined by the presence of signs and symptoms such as chest pain), both males and females, between 35 to 65 years, and presenting within 24 hours of the onset of symptoms and having normal ECG reports were included. Normal ECG reports were defined as reports that demonstrated no remarkable features, including ST-segment alterations and/or T wave inversion, amongst other alterations. The included age interval (35-65) was chosen considering that most patients presenting to the Tabba Heart Institute (THI) with ACS fall within that range.

Exclusion criteria

Known cases of valvular heart disease, cardiomyopathy, and congenital heart diseases were excluded. These factors were excluded to ensure that any alterations in the ECG findings were exclusively because of ACS and not, in fact, due to confounding variables such as valvular abnormalities.

The data obtained was entered and analysed using the SPSS version 22.0 (IBM Corp., Armonk, NY). Qualitative variables, such as gender, hypertension, diabetes, dyslipidemia, smoking status, obesity, family history, and NSTEMI, were presented as frequencies and percentages. Quantitative variables, such as age and duration of symptoms, were expressed in terms of Mean ± SD. Effect modifiers like age, gender, diabetes, hypertension, smoking status, dyslipidemia, and obesity were addressed through stratification to see the effects of these variables on the outcome. Post-stratification, the Chi-square test was applied; a p-value of 0.05 or less was considered significant.

## Results

**Figure 1:**
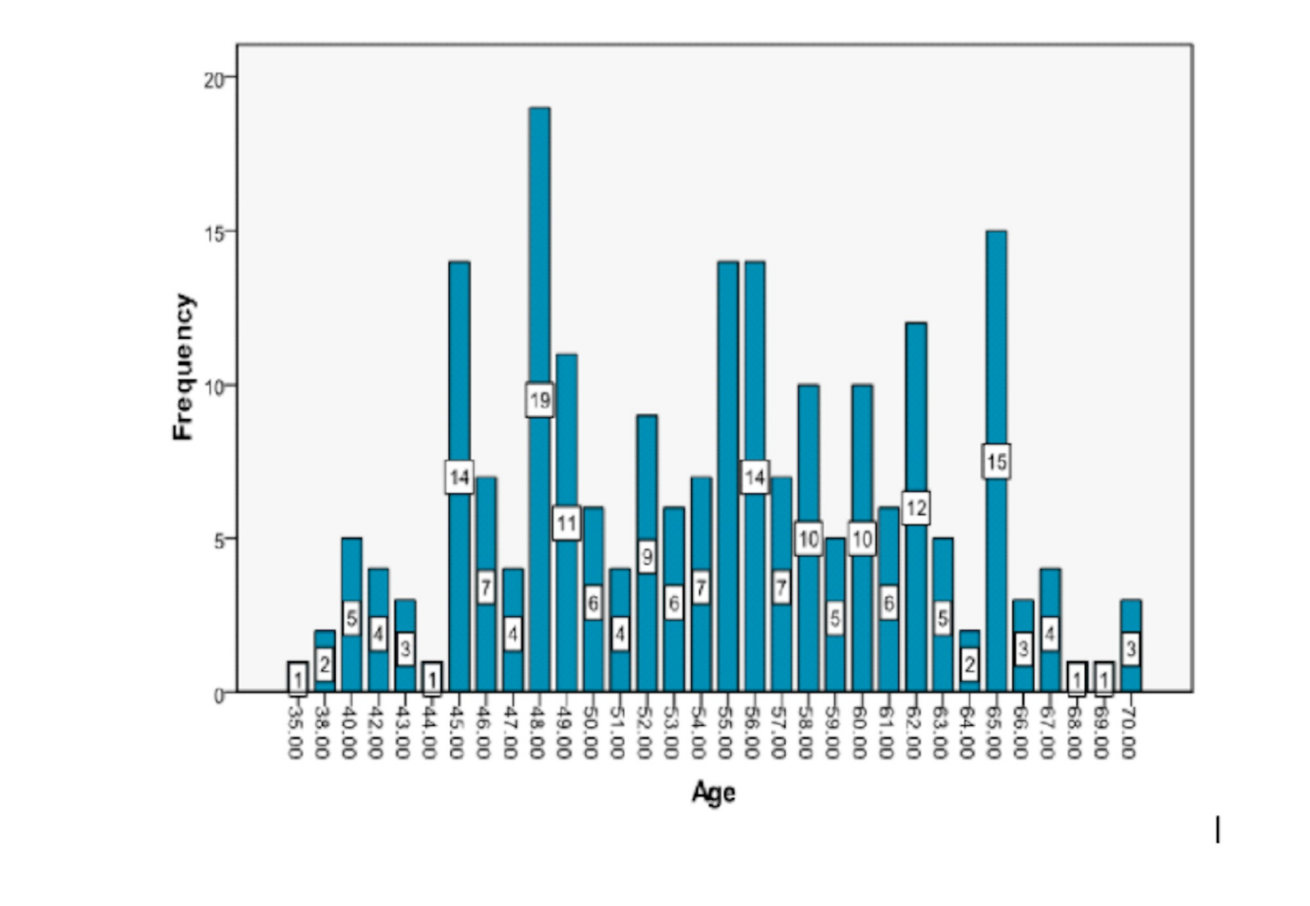
Frequency distribution of age (years)

A total of 215 patients, evaluated over one month, with ACS presenting within 24 hours of the onset of symptoms having normal ECG were selected for the purposes of this study. The mean age of the patients was 54.3 + 7.592 years. The distribution of age is presented in Figure 1below, along with the descriptive statistics of age as they relate to the occurrence of NSTEMI (presented in Table 1).

**Table 1:** Non-ST Segment Elevation Myocardial Infarction according to age (years) A Chi-square test was applied and a P-value ≤ 0.05 was considered significant.

Age (years)	Non-ST Segment Elevation Myocardial Infarction		Total	Odds Ratio	P-value
	Yes	No			
35-52 years	7 (3.29%)	85 (22.56%)	92 (25.85%)	6.300	0.007
53-65 years	42 (19.51%)	81 (54.64%)	123 (74.15%)		
Total	49 (22.8%)	166 (77.2%)	215 (100%)		

Of the 215 patients, 138 (64.2%) were males and 77 (35.8%) were females. The mean duration of the symptoms was noted to be 9.166 + 4.500 hours. The distribution of the duration of symptoms is presented in Table 2, along with the descriptive statistics of the duration of symptoms, such as troponin I levels and BMI. The mean BMI was 32.441 + 2.944 kg/m^2^; the descriptive statistics pertaining to BMI are also presented in Table 2. The mean troponin I level was 301.594 + 990.323 pg/ml and the mean change in troponin I levels was 202.715 + 653.056 pg/ml (shown in Table 2). In our study, diabetes mellitus (DM) was seen in 102 patients (47.4%) while hypertension (HTN) was seen in 100 (46.5%) patients, as shown in Table 3. A positive smoking history was noted in 79 patients (36.7%) and dyslipidemia was seen in 19 (8.8%) patients, as tabulated in Table 3. Imperatively, a family history of MI was present in 35 patients (16.3%). It is essential to note that in our study, non-ST segment elevation myocardial infarction (NSTEMI), as confirmed by a meticulous evaluation of cardiac biomarkers, was seen in 49 patients (22.8%).

**Table 2:** Descriptive statistics pertaining to age, duration of symptoms, BMI, troponin I levels, delta troponin I level (pg/ml)

Statistics	Age (Years)	Duration of symptoms (hours)	BMI (kg/m^2^)	Troponin I level (pg/ml)	Delta Troponin I level (pg/ml)
Minimum	35	2	26	1	0.80
Maximum	70	22	38	9300	4125
Mean	54.325	9.166	32.441	301.594	202.715
Std. Deviation	7.592	4.500	2.944	990.323	653.056

The frequencies of age groups, gender, duration of symptoms, DM, HTN, dyslipidemia, obesity, smoking, and a family history positive for MI were calculated; the results are presented in Table 3. It is noteworthy that in our study, NSTEMI was significantly associated with DM, hypertension, and age with p-values of 0.001, 0.001, and 0.007, respectively. It is equally important to highlight, however, that episodes of NSTEMI were not significantly associated with gender, duration of symptoms, dyslipidemia, obesity, smoking, or a family history indicative of MI (p-values: 0.599, 0.344, 0.339, 0.167, 0.311 and 0.080, respectively).

**Table 3:** Frequency distribution of gender with respect to baseline characteristics

Gender	Frequency (n)	Percentage (%)
Male	138	64.2%
Female	77	35.8%
Total	215	100%
Diabetes mellitus	Frequency (n)	Percentage (%)
Yes	102	47.4%
No	113	52.6%
Total	215	100%
Hypertension	Frequency (n)	Percentage (%)
Yes	100	46.5%
No	115	53.5%
Total	215	100%
Smoking	Frequency (n)	Percentage (%)
Yes	79	36.7%
No	136	63.3%
Total	215	100%
Dyslipidemia	Frequency (n)	Percentage (%)
Yes	19	8.8%
No	196	91.8%
Total	215	100%
Family history of MI	Frequency (n)	Percentage (%)
Yes	35	16.3%
No	180	83.7%
Total	215	100%
Obesity	Frequency (n)	Percentage (%)
Yes	169	78.6%
No	46	21.4%
Total	215	100%

## Discussion

ECG is a useful tool for risk stratification of patients who present to the ED with chest pain. Studies during the past two decades have revealed that low-risk patients presenting with chest pain can be identified through a combination of clinical evaluation and ECG at the time of ED presentation [9-11]. Generally, a normal ECG is associated with low risk of cardiac complications and mortality. However, the ECG is imperfect in this regard due to its limited ability to detect ischemia in the distribution of the left circumflex coronary artery, in the true posterior left ventricular region, and in patients with prior acute myocardial infarction (AMI) [12]. ECG evidence of ischemia may also be transient, further deluding patient presentation [13].

Many patients with NSTE-ACS can have unremarkable ECG findings on presentation in the emergency room, showing that ECG may be non-diagnostic in ACS in many cases [14]. Patients presenting to the emergency department with chest pain and a normal ECG have a low rate of morbidity and mortality from cardiac complications. Regardless, these complications are not negligible and should therefore borne in mind [15-17].

In our study, NSTEMI was seen in 49 patients (22.8%). A study by Turnipseed et al. showed that the frequency of ACS in patients with normal ECG was 17% [6]. Studies during the past decades have reported a 3% to 10% incidence of AMI in patients presenting to the ED with chest pain and a normal ECG [18, 19]. Additionally, Singer et al., employing creatine phosphokinase-myocardial band (CPK-MB) as a marker of cardiac injury, found that 17% of AMI patients initially presented with normal ECGs [18]. Using cardiac troponin I or CPK-MB, Forest et al., reported an AMI rate of 2% in 1,912 patients with chest pain and a normal ECG [19]. Interestingly, in a recent study evaluating cardiac troponin enzymes and their implications in cardiac injury, Chase et al. found a 2.8% frequency of AMI in ED patients with a normal or nonspecifically altered ECG [6]. Our data demonstrates a higher incidence of AMI (7%) than the studies mentioned above. This finding is likely influenced by the fact that 116 patients underwent immediate exercise treadmill testing after evaluation of only one negative cardiac marker and were discharged after a negative test.

Risk stratification is a crucial step in ACS, with significant implications on patient management and prognosis. Many risk scores and factors have been published in medical literature during the last decade to help attain a more precise clinical decision. Historically, ECG has been pivotal in evaluating electrophysiological disturbances underlying cardiac pathology, but physicians should be wary of their limitations.

A study by Teixeira et al., further observed that 22% of patients with a normal ECG went on to develop an NSTEMI, which on a practical level forces the clinician to achieve a precise clinical and biochemical characterisation, considering that a normal ECG may not necessarily be a benign finding. We believe that ischemic changes are dynamic and can thus evade detection [20].

Our study, however, is replete with limitations. Our study, for instance, was a single-center study, with a relatively small sample size. This means that the results obtained from our study cannot readily be generalised. Further studies with larger sample sizes are therefore required to truly ascertain the predictive ability of ECG. Furthermore, in hospitals experiencing an exorbitant influx of patients, the evaluation of cardiac enzymes might not always be feasible, meaning that a multitude of factors, included the associated costs, ought to be considered.

## Conclusions

The frequency of NSTEMI was high among ACS patients presenting within 24 hours of the onset of symptoms on a background of unremarkable ECG findings. Unremarkable ECG findings should be construed with caution and thus categorically complemented with the evaluation of cardiac enzymes to conclusively ascertain the absence of an NSTEMI.
